# Anterior Cruciate Ligament Tear: Individualized Indications for Non-Operative Management

**DOI:** 10.3390/jcm13206233

**Published:** 2024-10-18

**Authors:** George A. Komnos, Michael H. Hantes, Georgios Kalifis, Nifon K. Gkekas, Artemis Hante, Jacques Menetrey

**Affiliations:** 1 Department of Orthopaedic Surgery and Musculoskeletal Trauma, University Hospital of Larissa, 41110 Larissa, Greece; 2 Department of Orthopaedic Surgery and Musculoskeletal Trauma, University Hospital of Larissa, School of Health Sciences, University of Thessaly, 41110 Larissa, Greece; 3 Minimally Invasive Surgery Orthopaedic Center, St. Luke’s Hospital, 55236 Panorama, Greece; 4 Physiotherapy Department, International Hellenic University, 57001 Nea Moudania, Greece; 5 Centre de Médecine du Sport et de L’Exercice—Swiss Olympic Medical Center, Hirslanden Clinique La Colline, 1206 Geneva, Switzerland

**Keywords:** ACL rupture, individualized approach, conservative treatment, surgery, knee, sport

## Abstract

Anterior cruciate ligament (ACL) rupture represents a common sports injury that is mostly managed operatively. However, non-operative treatment can also play a role, despite the limited high-quality published data on ACL tear management. Both methods have shown favorable outcomes, but clear guidelines based on high-quality research are lacking. Several factors should be considered and discussed with the patient before deciding on the best treatment method. These include patient characteristics and expectations, concomitant injuries, and clinical evaluation, with laxity or/and instability being one of the most essential parameters examined. This should eventually lead to an individualized approach for each patient to ensure the best possible outcome. This review aims to delve into all parameters that are related to ACL rupture and guide physicians in choosing the most appropriate treatment method for each patient.

## 1. Introduction

Anterior cruciate ligament (ACL) injuries represent one of the most common sports-related knee injuries. These occur most frequently during sports activities, especially those involving twisting and cutting movements [1,2]. The management of ACL injuries is challenging due to the prolonged absence of sports activity and prolonged rehabilitation periods. Additionally, there is a potential predisposition to post-traumatic osteoarthritis (PTOA), which may negatively impact the patient’s activity level and quality of life [3,4].

Because operative treatment has complications, which may be rare but serious, it is crucial to avoid surgery when it is not necessary. Patient characteristics such as age and gender, sports and general activity, and concomitant injuries should be considered before deciding on operative or conservative management. One of the main issues in managing ACL rupture is the low quality of published data, particularly those comparing the two methods (operative and non-operative). Recent randomized controlled trials (RCTs) focus on specific populations and cannot provide general recommendations [5].

Currently, both operative and non-operative management are acceptable methods for treating ACL injuries. The primary objectives are to restore knee stability, prevent secondary meniscal and cartilage damage, and ultimately avoid post-traumatic osteoarthritis [3,4,5]. Several effective techniques have been proposed and established for surgical treatment. In conservative treatment, the neuromuscular system plays the most significant role in restoring muscle capacity and functional knee stability [6,7]. Neuromuscular rehabilitation focuses on training and educating the remaining restraints, especially the hamstring muscles.

This review aims to give an up-to-date insight into current literature data regarding ACL injury management and provide useful information that may guide the orthopedic surgeon and/or sports medical doctor and the patient to the best individualized approach. A thorough review and evaluation of the available literature until the end of 2023 was performed. The inclusion criteria consisted of all non-pediatric patients undergoing conservative treatment for ACL rupture, including rehabilitation, physical therapy, and non-operative management, as well as all non-pediatric patients undergoing operative treatment for ACL rupture, specifically ACL reconstruction surgery. Studies reporting patient satisfaction, and studies evaluating the incidence or progression of osteoarthritis, were also integrated. All types of studies were encompassed, such as randomized controlled trials (RCTs), cohort studies, case–control studies, and observational studies, with a minimum follow-up period of 12 months to assess sufficient outcomes. Only studies published in English, until the end of 2023, with full-text articles available were reviewed. The exclusion criteria were studies with patients with multi-ligament knee injuries (beyond isolated ACL rupture), those focusing solely on surgical techniques without comparing conservative treatments or addressing acute management (within the first 6 weeks post-injury), those without long-term follow-ups, and those written in languages other than English.

## 2. Epidemiology and Different Profiles of Patients

Isolated ACL injuries represent almost half of all knee ligament injuries [8]. Their incidence has been reported as being up to approximately 85 per 100,000 in younger, more active patients [9,10]. More than 200,000 ACL reconstructions are estimated to have taken place in 2014 in the USA, while 50,187 reconstructions were reported between 2003 and 2008 in Australia [11,12]. As expected, the incidence of ACL reconstructions is lower and notably different among various countries. In the UK, it is reported to be about 13.5 per 100,000 [13]; in Australia, 52 per 100,000 [12]; and in Scandinavian countries, 38 per 100,000 [14]. In a study including patients from a tertiary health care system, it was found that less than one-quarter of patients who were diagnosed with an ACL tear eventually underwent reconstruction in the following 3 years [15]. A recent analysis of knee injury trends in Australia reported that the number of ACL injuries had the fastest annual growth, especially in the pediatric population. The total number of ACL injuries is expected to more than double by 2030 [4].

Evaluating the true impact of ACL injuries may be challenging. Although there has been a great number of studies focusing on the outcomes of operatively treated patients, conservatively treated ACL injuries may be under-reported [16]. In general, most data are extracted from registries and subsequently mainly represent surgical cases [14,17]. Studies based on institutional data have shown that the actual rupture rate can be 40% higher than the surgical incidence [18]. In a nationwide study, it was reported that the incidence of ACL reconstructions was almost half of the total incidence of ACL ruptures: 39.4 and 75.1 per 100,000, respectively [19].

Different patient patterns may also exist among the population. It is known that males present peak incidence in their early twenties [19,20]; on the contrary, females are reported to show two peaks, one in their teens and another in their forties [19]. This may be due to the fact that many women have to take a partial pause from sports activities during childrearing, resuming such activities later. Parsons et al. suggested that gender in ACL injuries should be approached as an extrinsic social construct as well, rather than only from a biological perspective [21]. On the other hand, the decrease in ACL injuries during aging in men is probably indicative of a decline in high-risk sports participation. Seil et al. revealed that males are more prone to injuries during pivoting or contact sports before the age of 35, while females are more prone to them during recreational skiing at the age of 35 [22].

## 3. Patient Characteristics

The patient characteristics that can influence the final decision about the preferred treatment remain under investigation. In one of the most famous and widely used approaches, three different types of patients suffering from ACL rupture are proposed [23,24]: (1) the coper, who can return to sports at an acceptable level, equivalent to the pre-injury one, without surgical reconstruction; (2) the adapter, who returns to a reduced activity level to avoid instability symptoms; and (3) the non-coper, who is eventually not able to resume sports activity due to instability. Fitzgerald et al. developed a quite reliable tool in an attempt to identify copers and non-copers [25]. This included a combination of hop tests and questionnaires about knee function and subjective instability. This tool, in conjunction with the other parameters that are discussed in this review, can be used to identify the most appropriate candidates for operative treatment.

According to some data, gender and age are not significant predictive factors for surgery [26,27]. Van der Graaff et al. demonstrated that those who failed non-operative treatment and subsequently underwent ACL reconstruction were significantly younger [28]. Age-related differences in the incidence of ACL rupture have been found between males and females. Females tend to have an early initial peak in their late teens and another peak in their forties, while men reveal only one peak in their twenties [19]. However, significant annual growth in the rate of female ACL injuries, especially in adolescence, has recently been reported [21]. Interestingly, the majority of females from the second peak are not treated surgically.

As the majority of young patients are eventually led to surgery, some may argue that older patients should be treated conservatively. In a Swedish study, almost half of injured patients between 21 and 30 years old underwent reconstruction [29]. Furthermore, the percentage of patients undergoing reconstruction decreased by about 10 percent every decade. However, the number of patients aged 40 years or older who have their ACL reconstructed is constantly rising, and age is no longer considered an absolute risk factor against operative treatment, especially in active patients [30]. Subsequently, when considering age, we have to evaluate physical activity as well. Non-operative treatment may result in satisfactory results in middle-aged patients with low athletic demands, but secondary instability may prevent a return to the desired athletic level activity [31]. Corona et al. came to the conclusion that there is no significant difference in the outcomes of ACL reconstruction in patients <50 or >50 years old [32], while Salesky et al., in their large cohort study of more than 20,000 patients, concluded that patients >50 years old have a significantly higher risk of medical complications, while younger patients have a higher risk of a subsequent knee surgery [33]. Interestingly, recent studies have reported similar outcomes of ACL injury management in both younger and older patients [34]. Seng et al. demonstrated that the benefits of surgical treatment outweigh the risk of surgical complications in patients aged 40 years or older [35]. Fayard et al. reported satisfactory clinical and functional results following ACL reconstruction in over-50-year-old patients [36]. However, they highlighted that patient selection is extremely important, as radiologic evidence of osteoarthritis in the medial compartment can lead to inferior outcomes. The same conclusion about over-50-year-old patients was also previously supported by Dahm et al. [37]. Both studies proposed that operative management is the optimal strategy for these cases, especially for those who wish to take part in physical activity that includes pivoting. Of note, similar results have also been presented even for patients over 60 years old [38], thought the presence of osteoarthritis or limb malalignment may be a limiting factor [39,40].

## 4. Clinical Evaluation and Imaging

There is always the necessity for accurate physical examination to avoid non-essential operative treatment. A clinical evaluation of residual laxity is extremely important. A complete physical examination of the knee that provides the highest sensitivity and specificity for an ACL tear contains the Lachman test, the pivot shift, and the anterior drawer test [41]. However, these tests may have high variability among examiners, and the pivot shift test, in particular, can be difficult to perform [42,43]. Different measurement devices have been developed so as to objectively assess anteroposterior and rotational knee laxity. Several objective measurement systems exist to assess anteroposterior (AP) laxity [43,44]. It is suggested that the KT-1000 knee arthrometer, the Rolimeter, and, recently, the GNRB arthrometer provide the best results when testing anterior knee laxity [45]. Recently, new devices such as the Rotameter have been introduced that are capable of assessing rotational laxity as well [46]. Martinez-Cano et al., in their systematic review, concluded that instrument-based assessments of laxity have high intra-observer and inter-observer reliability [47]. The utilization of both instrumented anterior and rotational knee laxity measurements can capture all ACL ruptures and evaluate the clinical instability, which assists with identifying the preferred means of treatment [48]. The GNRB arthrometer can automatically assess both anterior and rotational laxity with high accuracy by applying a standardized force [48].

Clinical evaluations and the utilization of the above-mentioned devices are intended to assess instability and laxity and guide treatment. Mild instability (defined as Lachman grade 1) can be treated conservatively based on published data [49,50]. In patients with mild instability, Ahn et al. showed that knee laxity on clinical examination was improved at the last follow-up [49], with the Lachman test improving to grade 0 in 87% of the patients and 76% of them having a negative pivot shift test. In their cohort, there were 12 complete (25%) and 36 incomplete ACL ruptures (75%). A randomized study comparing surgical and non-surgical treatment for ACL injury revealed that more than half of young, active people could have very good outcomes even without operation when no symptomatic knee instability occurs [51]. Park et al. reported a 91% success rate in patients with pivot shift and/or Lachman test grade 1 who were treated non-operatively [50].

Daniel et al. identified side-to-side differences measured with the KT-1000 arthrometer as a reliable tool to predict late meniscus or ligament surgery [27]. They classified a difference of 3 mm or greater between the injured and non-affected knees as indicating a KT unstable knee. Moreover, negative Lachman and pivot shift tests, along with a KT-1000 side-to-side difference of less than 3 mm at 6–12 weeks after the injury, can predict good functional outcomes, as evaluated by the IKDC subjective score, and normal knee anterior laxity at more than 2 years in recreational alpine skiers [52].

Besides laxity, clinical evaluations with single, one-leg hop tests and quadriceps femoris maximum voluntary isometric strength tests, which can reveal a potential muscle and joint imbalance, should also be performed in an attempt to identify potential copers [25,53,54]. Furthermore, detailed history taking is essential to evaluate if the patient reports giving-way symptoms, which are indicative of instability [25,54]. Symptomatic instability, impaired knee function, and pain during daily activities have been reported to be factors leading to delayed ACL reconstruction after conservative management failure [28]. Giving way and recurrent swelling were also considered as significant determinants in favor of surgical treatment among American orthopedic surgeons [55,56].

Regarding imaging, MRI findings are not completely accurate, with a sensitivity of 81% and a specificity of 96% [56]. Though MRI findings may be indicative of a loss of ACL continuity, physical examinations and measurements of laxity can identify cases where there is still some ACL continuity. Particular MRI findings, such as retained continuity of a swollen ACL with a relatively low signal intensity, can predict a high probability of success of non-operative treatment. Moon et al. concluded that anterior tibial translation and the extent of bone bruises were predictive of failure of non-operative management in partial ACL tears [57].

In general, the findings of an MRI scan may be indicative of a partial tear, but these cannot be applied to make a definitive diagnosis since MRI has low sensitivity and specificity for diagnosing partial tears [8]. Its utility mainly applies in identifying concomitant intra-articular lesions [41].

In conclusion, clinical and imaging evaluations are extremely important, as the orthopedic surgeon can gather and evaluate all the available findings and identify those patients who will benefit from surgery.

## 5. Sports Activity

The available literature tends to propose that patients with high athletic demands or who wish to keep on with pivoting sports at a satisfying level should be managed operatively [58]. In addition, their age and previous physical activity level should be considered before decision-making.

A high activity level prior to injury, expressed as 8 or 9 on the Tegner scale, is proved to predispose patients in favor of ACL reconstruction [26]. The pre-injury level of practice is also essential [22]. Typically, athletes who desire to return to pivoting sports are counseled to undergo surgery, since it provides good clinical outcomes and allows most of them to return to their pre-injury level [59,60]. Fink et al. evaluated the long-term outcomes of operative or non-operative treatment and identified sports activity as a determining variable [61]. They found that the patients who were treated operatively evaluated themselves better and maintained higher levels of sports participation. Seil et al. found that the percentage of surgical treatment was superior to 80% in patients less than 35 years of age involved in competitive sports [22].

However, opposing results have also been presented. Grindem at al., in a pair-matched comparison of return to pivoting sports at 1 year, indicated no significant difference in the overall return to sport rates or in the return to level I sports between operative and non-operative treatments [62]. Still, the percentage of non-operatively treated patients who returned to level I sports was statistically significantly lower than the percentage of those who participated in level II sports (54.8% and 88.9%, respectively). Furthermore, in two comparative studies, patients who were treated non-operatively had a higher return rate to level II and III sports than did those who received operative treatment [62,63]. Grenverts et al. suggested that in non-high-demand patients, initial non-operative management for >3 months may aid in the identification of copers and guide further management with delayed reconstruction, if necessary [31]. We can conclude that in a return to straight-plane activities, non-operative treatment can be an option [64].

## 6. Associated Lesions

Concomitant lesions during the initial injury can play an essential role in patients’ management of ACL tears. Farinelli et al., in their study including elite alpine skiers and professional soccer players with ACL injuries, reported meniscal tears in up to 83% and chondral injuries in up to 34% of the patients. They also concluded that professional soccer players were more likely to sustain medial meniscus and lateral meniscal root tears [65]. It has been reported that when a ramp lesion of the medial meniscus co-exists in the setting of an ACL injury, the dynamic rotational laxity in the injured knee is higher, with a higher grade in the pivot shift test [66]. This should always be taken into consideration, since it should not be misdiagnosed, and it can also guide treatment to repair it along with the ACL reconstruction. D’Ambrosi et al., in their systematic review, concluded that all unstable ramp lesions in the ACL injury setting should be repaired, while this is yet unclear for stable lesions [67]. Meniscus lesion management performed simultaneously with ACL reconstruction has shown superior outcomes with repair rather than resection [68]. This was further validated by a recent systematic review, which pointed out that the repair of meniscal lesions results in better anterior knee joint stability with better patient-reported outcomes, but with the drawback of higher re-operation rates that reach more than 13% in comparison to less than 1% in meniscectomies [69].

It is widely accepted that the elimination of the hazard of secondary knee injuries, including meniscal tears and cartilage damage, is of paramount importance after an ACL injury. In the question of whether surgical treatment reduces the risk of subsequent lesions, a recent systematic review could not make a concrete conclusion in favor of surgical treatment compared with treatment with rehabilitation only [70]. Nonetheless, Sanders et al. found that non-operated patients had a higher risk of secondary meniscal tear and proposed that ACLR significantly reduces the risk of subsequent injury to the menisci [71]. Moreover, Yoo et al. stated that delayed reconstruction heightens the likelihood of a medial meniscal tear [72]. Giurazza et al., in their analysis of 4697 knees, concluded that almost 40% of the patients undergoing ACL reconstruction had associated medial meniscus tears, and they identified high BMI, male sex, and lateral meniscal tears as secondary injury risk factors. Additionally, they highlighted that the incidence and complexity of the tears increased significantly as the time from the injury to the operation increased [73]. Noteworthily, although associated meniscal injuries have been widely investigated, data are scarce regarding cartilage injuries. More specifically, associated cartilaginous lesions are very difficult to identify by clinical examination or imaging. On the contrary, this can be performed much more easily during arthroscopic examinations in cases of surgical management.

## 7. Reported Outcomes

In general, it is widely reported that patient-reported knee function and muscle strength are equivalent in both operated and conservatively treated patients [52,63,74,75]. Gföller et al. demonstrated good subjective knee function after 20 years of ACL deficiency [76]. Although increased joint laxity and arthritic changes were observed at the end of the follow-up period, patient satisfaction, expressed by subjective International Knee Documentation Committee (IKDC) scores and ratings of knee function, was well maintained after over 20 years of follow-up, with a gradual improvement over time. In another retrospective study that compared operative versus non-operative treatment in high-level athletes, besides better knee stability in the operative group, no difference was found in subjective and objective functional outcomes [77]. In a cross-sectional study based on the Swedish knee ligament registry, it was demonstrated that patients from the ACL reconstruction group had better knee function and quality of life compared to the non-operative management group at follow-up of up to 10 years [78]. Ardern et al. reported superior outcomes in patients who had chosen ACL reconstruction, as expressed through the KOOS (Knee Injury and Osteoarthritis Outcome) and EuroQoL-5 D questionnaires [79]. On the contrary, a recent systematic review was unable to identify the superiority of either management type in terms of the reported PROMs expressed through the IKDC, Tegner, KOOS, and Lysholm scores [80]. Macri et al., in their systematic review, suggested that PROMs in ACL injuries are great tools for interpretability in clinical practice and research; however, they should be evaluated with caution due to the high heterogeneity among published studies [81].

ACL reconstruction was previously considered the only viable solution for avoiding post-traumatic arthritis following ACL rupture. Nevertheless, we are currently aware that ACL reconstruction does not prevent knee OA and that post-traumatic OA can develop independently of the management of the ACL rupture. In this context, combined lesions are the most important ones [82,83]. Published data are extremely conflicting as to whether ACL reconstruction prevents or at least reduces the incidence of knee OA following ACL rupture. ACL rupture itself predisposes the knee to a 4-fold increase in risk compared to the non-injured knee and a 6-fold increase in risk compared to the general population [84,85]. A decade after sustaining an ACL injury, almost half of patients have radiographic signs indicative of OA [83,86,87,88]. Therefore, it can be assumed that the fate of the knee is more or less defined by the initial injury. In an up to-date systematic review, Lie et al. demonstrated that the OA prevalence at 10 years post-ACL rupture does not significantly differ between those treated surgically (8–68%) and non-surgically (24–80%) [82]. In another recent meta-analysis, the prevalence of radiographic knee OA was lower in the group of patients who received non-surgical treatment [80]. On the contrary, in a prospective cohort study, patients who underwent early ACL reconstruction had a lower prevalence of tibiofemoral radiographic OA than those who did not (50% vs. 75%) [89]. Liukkonen et al., in their systematic review, reported that around 40% of the patients with an ACL injury developed post-traumatic knee OA at a 15-year follow-up, without significant differences between reconstruction and conservative management [90]. Everhart et al. concluded that the prevalence of moderate to severe post-traumatic OA after ACL reconstruction was 25% at a minimum of 20 years’ follow-up. They also highlighted that medial meniscectomy was associated with a higher risk of OA development, although the grading of the knee OA was associated with impaired knee function but not with knee pain [91].

Besides osteoarthritis, the relationship between reconstruction or not of the ACL and secondary intra-articular lesions, predominantly meniscal tears, has been widely investigated. Many authors argue that chronic micro-instability after an ACL tear gradually results in new meniscal lesions [92,93]. Sanders et al. reported that patients who did not have their ACL reconstructed had a significantly higher risk of secondary meniscal tears [94]. In another study, the presence of cartilage lesions was remarkably increased in the ACL-deficient knee, but no significant increase in the incidence of new meniscal tears was reported [95].

In a matched-pair analysis of high-level athletes with an average of ten years’ follow-up, no difference was indicated between operated and non-operated athletes in the setting of numerous parameters [75]. These included osteoarthritis, meniscal lesions, activity levels, and objective and subjective functional outcomes. Patient-reported outcomes were found to be similar at 2- and 5-year follow-ups among 121 young, active patients with isolated ACL tears in a randomized controlled trial, where two groups of operative and non-operative or delayed operative treatment were evaluated [52]. Nevertheless, a high percentage of the patients who were treated non-operatively required delayed ACL reconstruction, and almost one-third of them finally underwent an operation for meniscal injuries during the 2-year follow-up period. Keays et al. studied two groups of operative versus non-operative management of ACL injuries and concluded that patients who underwent reconstruction were 3 times more likely to return to competitive pivoting sports [96].

## 8. Discussion

Both operative and non-operative treatments of an ACL injury are developing in favor of patients’ knee function and reported outcomes. Reconstruction of the ACL is considered a so-called routine procedure with satisfactory outcomes. Nonetheless, complications may occur, leading to further surgeries [97]. Although rare, these include arthrofibrosis, infection, graft failure, donor site morbidity, and pain. Surgical costs deriving from the operative intervention could also be considered a factor in favor of non-surgical management [98]. Some studies strived to estimate the cost-effectiveness of surgical or non-surgical treatment, but no concrete conclusions were derived. This lies in the wide variety of different parameters and factors that are involved in both means of treatment, which makes a direct assessment inevitable [98,99]. It has been recently suggested by various authors that early ACL reconstruction might be cost-effective only in young, high-demand populations. In addition, rehabilitation plus optional delayed reconstruction in certain groups of patients that do not respond to conservative management may be a more pragmatic approach [100,101].

Even though the literature is rich in data about ACL injury management, no concrete guidelines exist. Several efforts have been made by orthopedic societies or groups of experts to provide guidelines for ACL management [10,41,64]. They mostly agree that the published data are insufficient and there is a need for large randomized clinical trials with long-term follow-up comparing surgery with rehabilitation. Indeed, randomization could be the only way to undertake a high-quality study for the most reliable indications for ACL reconstruction [102]. Petersen et al., in their consensus project, proposed an algorithm for ACL injury management and concluded that although there is evidence to support this treatment strategy, higher-quality studies are needed to substantiate it [103].

Of paramount importance is to underline the true incidence of ACL ruptures, which may be higher than previously reported. Besides the ACL reconstruction registries, records of ACL-injured patients do not yet exist. Therefore, the best way to evaluate the true incidence of ACL ruptures is by adopting an ACL rupture registry, which could be of considerable help in guiding treatment in specific subgroups of patients, where controversy remains [104]. National registries will also assist in identifying the varying patient characteristics, which differ from country to country. The standardization of data collection and the potential benefits of artificial intelligence use to create decision-making tools should be the future direction of ACL registries and related research [16].

Many nonidentical profiles of patients exist, and knowledge of their specific features may improve our judgment of the best applied individualized approach. For instance, nowadays, unlike in previous years, many middle-aged and elderly people remain active, which has led to an increased rate of injuries in this group of patients who then experience problems returning to a desirable pre-injury activity [105]. Conservative treatment was previously reserved for these cases, but this is not an absolute rule nowadays [33].

**Figure 1:**
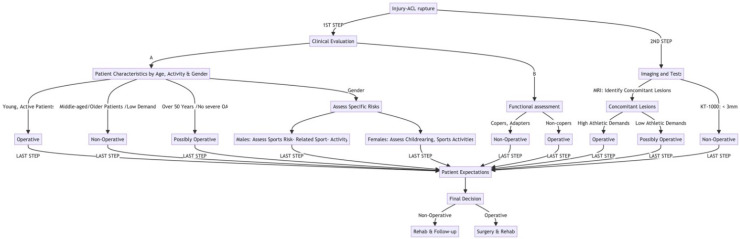
Our proposed algorithm for dealing with ACL ruptures.

The orthopedic surgeon has to take into consideration all the parameters that are analyzed in the current review and present them to the patient. All these parameters, along with the patient’s expectations and goals and the clinical course that will follow either option, should be discussed, and a joint decision between the physician and the patient must always be made (Figure 1). Our proposed algorithm for ending up at a decision regarding the management of ACL rupture incorporates all the available data that are presented and analyzed above; our belief is that it could be a powerful tool in the hands of all physicians dealing with these injuries (both sports medicine doctors and orthopedic surgeons).

## 9. Conclusions

The identification of those patients who are appropriate for operative or non-operative treatment is of utmost importance. Copers or adaptors, who can return to their previous activity without significant impairment, and patients with no subjective or objective instability are, in general, good candidates for conservative functional treatment. On the contrary, patients with persistent laxity and/or high demands regarding sports activities should mostly be treated with ACL reconstruction. Other existing factors, such as the presence of osteoarthritis and concomitant injuries, should always be assessed as well. In the end, it lies to the physician to evaluate the available data, which are mainly based on general evidence, and apply this generality to individuals.
